# Landscape of Familial Isolated and Young-Onset Pituitary Adenomas: Prospective Diagnosis in *AIP* Mutation Carriers

**DOI:** 10.1210/jc.2015-1869

**Published:** 2015-07-17

**Authors:** Laura C. Hernández-Ramírez, Plamena Gabrovska, Judit Dénes, Karen Stals, Giampaolo Trivellin, Daniel Tilley, Francesco Ferraù, Jane Evanson, Sian Ellard, Ashley B. Grossman, Federico Roncaroli, Mônica R. Gadelha, Márta Korbonits

**Affiliations:** Centre for Endocrinology (L.C.H.-R., P.G., J.D., G.T., D.T., F.F., J.E., M.K.), William Harvey Research Institute, Barts and The London School of Medicine, Queen Mary University of London, London EC1M 6BQ, United Kingdom; Department of Molecular Genetics (K.S., S.E.), Royal Devon and Exeter National Health Service Foundation Trust, Exeter EX2 5DW, United Kingdom; Program on Developmental Endocrinology and Genetics (G.T.), Section on Endocrinology and Genetics, *Eunice Kennedy Shriver* National Institute of Child Health and Human Development, National Institutes of Health, Bethesda, Maryland 20892; Department of Endocrinology (A.B.G.), Oxford Centre for Diabetes, Endocrinology, and Metabolism, Churchill Hospital. Headington, Oxford OX3 7LE, United Kingdom; Division of Brain Sciences (F.R.), Faculty of Medicine, Charing Cross Hospital, Imperial College, London W6 8RP, United Kingdom; and Endocrinology Unit (M.R.G.), Clementino Fraga Filho University Hospital, Federal University of Rio de Janeiro, Rua Professor Rodolpho Paulo Rocco, Ilha do Fundaõ, Rio de Janeiro 21941–913, Brazil; Beaumont Hospital, Dublin, Republic of Ireland; St Bartholomew's Hospital, London, United Kingdom; Barts and The London School of Medicine, Queen Mary University of London, London, United Kingdom; Szent Imre Egyeteni Oktatókórház, Budapest, Hungary; University of Birmingham, Birmingham, United Kingdom; Royal Victoria Hospital, Belfast, Northern Ireland, United Kingdom; Hospital de Sant Pau, Universitat Autònoma de Barcelona, Barcelona, Spain; Kings College Hospital National Health Service Foundation Trust, London, United Kingdom; Cincinnati Children's Hospital Medical Center, Cincinnati, Ohio; Carol Davila University of Medicine and Pharmacy, Bucharest, Romania; University College London Hospital, London, United Kingdom; St George's University of London, London, United Kingdom; University of Michigan Medical Center, Ann Arbor, Michigan; Leicester Royal Infirmary, Leicester, United Kingdom; Hospital Universitario “12 de Octubre,” Madrid, Spain; St Bartholomew's Hospital, London, United Kingdom; Aberdeen Royal Infirmary, Foresterhill, Aberdeen, United Kingdom; Alder Hey Children's National Health Service Foundation Trust, Liverpool, United Kingdom; Royal Free and University College School of Medicine, London, United Kingdom; St George's Healthcare National Health Service Trust, London, United Kingdom; University of Erlangen, Erlangen, Germany; Meram School of Medicine, Konya Necmettin Erbakan University, Konya, Turkey; North West Thames Regional Genetics Service, Northwick Park Hospital, London, United Kingdom; Guy's and St Thomas' National Health Service Foundation Trust, London, United Kingdom; Imperial College Healthcare National Health Service Trust, London, United Kingdom; University of Newcastle, Newcastle, United Kingdom; Bab El Oued Teching Hospital, Algiers, Algeria; University of Keele, Stoke-on-Trent, United Kingdom; Royal Free National Health Service Foundation Trust, Barnet Hospital, Barnet, United Kingdom; Birmingham Women's Hospital, Birmingham, United Kingdom; Royal Victoria Hospital, Belfast, Northern Ireland, United Kingdom; University Hospitals Bristol Foundation Trust, Bristol, United Kingdom; University of Liverpool, Liverpool Merseyside, United Kingdom; Aarhus University Hospital, Aarhus, Denmark; Endocrinology Research Centre, Moscow, Russia; University Hospital Aintree, Clinical Sciences Centre, University of Liverpool, Liverpool, United Kingdom; Lister Hospital, Stevenage, United Kingdom; University College London Institute of Child Health, London, United Kingdom; University Hospital Southampton, Southampton, United Kingdom; Faculty of Medical and Human Sciences, University of Manchester and Central Manchester University Hospitals National Health Service Foundation Trust, Manchester, United Kingdom; Ribeirao Preto School of Medicine, University of Sao Paulo, Sao Paulo, Brazil; Università Cattolica del Sacro Cuore, Policlinico Universitario A. Gemelli, Rome, Italy; St Bartholomew's Hospital, London, United Kingdom; Postgraduate Institute of Medical Education and Research, Chandigarh, India; Endocrinology Research Centre, Moscow, Russia; Uppsala University Hospital, Uppsala, Sweden; Sutton Hospital, Sutton, United Kingdom; Lund University Hospital, Lund, Sweden; Waikato Hospital, Hamilton, New Zealand and Waikato Clinical School, University of Auckland, Hamilton, New Zealand; All Wales Medical Genetics Service, Glan Clwyd Hospital, Rhyl, United Kingdom; Department of Oncology, University College London Hospitals, London, United Kingdom; Elias Hospital, Carol Davila University of Medicine and Pharmacy, Bucharest, Romania; Luzerner Kantonsspital, Luzern, Switzerland; Derriford Hospital, Plymouth, United Kingdom; Northwest Pituitary Center, Oregon Health and Science University, Portland, Oregon; Columbia University College of Physicians and Surgeons, New York, New York; Charles R. Drew University of Medicine and Science, Los Angeles, California; University of Illinois at Chicago, Chicago, Illinois; Western University, Children's Hospital, London Health Science Centre, London, Ontario, Canada; Barts and The London School of Medicine, Queen Mary University of London, London, United Kingdom; Semmelweis University, Budapest, Hungary; Harvard Vanguard Medical Associates, Boston Massachusetts; Imperial College Healthcare National Health Service Trust, Hammersmith Hospital, London, United Kingdom; Health Center, Hungarian Defense Forces, Budapest, Hungary; Alder Hey Children's Hospital Eaton Road, Liverpool, United Kingdom; National Hospital for Neurology and Neurosurgery, Queen Square, London, United Kingdom; Hospital Durand, Buenos Aires, Argentina; Faculty of Medicine, Hacettepe University, Ankara, Sihhiye, Turkey; University of Cambridge and Cambridge Biomedical Research Centre, Addenbrooke's Hospital, Cambridge, United Kingdom; Gyor Hospital, Gyor, Hungary; University Hospitals of Leicester National Health Service Trust, Leicester Royal Infirmary, Leicester, United Kingdom; Karolinska University Hospital, Stockholm, Sweden; Royal Victoria Hospital, Belfast, Northern Ireland, United Kingdom; Barts and The London School of Medicine, Queen Mary University of London, London, United Kingdom, and Università Cattolica del Sacro Cuore, Policlinico Universitario A. Gemelli, Rome, Italy; Faculty of Medicine, Semmelweis University, Budapest, Hungary; School of Medicine, The University of Queensland, Brisbane, Queensland, Australia; Institute of Health Biosciences, The University of Tokushima Graduate School, Toskushima City, Japan; Guy's and St Thomas' Foundation Trust, Guy's Hospital, London, United Kingdom; Mediscan, Chennai, India; Laiko General Hospital, School of Medicine, National and Kapodistrian University of Athens, Athens, Greece; Lister Hospital, Stevenage, United Kingdom; Oxford Centre for Diabetes, Endocrinology, and Metabolism, Churchill Hospital, Oxford, United Kingdom; University Hospital Zagreb, and School of Medicine University of Zagreb, Zagreb, Croatia; Massachusetts General Hospital and Harvard Medical School, Boston, Massachusetts; Greater Manchester Neurosciences Centre, Salford Royal Foundation Trust, Manchester, United Kingdom; University College London, London, United Kingdom; King Edward Memorial Hospital for Women, Subiaco, Australia; Medical Academy, Lithuanian University of Health Sciences, Kaunas, Lithuania; Flór Ferenc Hospital, Kistarcsa, Hungary; Great Ormond Street Hospital, London, United Kingdom; Brigham and Women's Hospital, Boston, Massachusetts; Tupper Research Institute, Tufts Medical Center, Tufts University School of Medicine, Boston, Massachusetts; University Hospitals of Leicester National Health Service Trust, Leicester Royal Infirmary, Leicester, United Kingdom; Polish Mother's Memorial Hospital-Research Institute, and Medical University, Lodz, Poland; Harvard Medical School, Massachusetts General Hospital, Boston, Massachusetts; University of Brisbaine, Brisbaine, Australia; Teikyo University, Tokyo, Japan; Guy's and St Thomas' Foundation Trust, St Thomas' Hospital, London, United Kingdom; Mater Misericordiae University Hospital, Dublin, Republic of Ireland; Clinical Center of Vojvodina and Medical Faculty, University of Novi Sad, Novi Sad, Serbia; Hospital de Especialidades Centro Médico Nacional Siglo XXI, Instituto Mexicano del Seguro Social, Mexico City, Mexico; Faculty of Medicine, University of Pécs, Pécs, Hungary; Clinical Center of Serbia and Medical Faculty, University of Belgrade, Belgrade, Serbia; Massachusetts General Hospital, Harvard Medical School, Boston, Massachusetts; Hospital das Clinicas, Minas Gerais Federal University, Belo Horizonte, Brazil; Northwestern University Feinberg School of Medicine, Chicago, Illinois; St Bartholomew's Hospital, London, United Kingdom; The Ipswich Hospital, Ipswich, United Kingdom; Belfast Health and Social Care Trust, Belfast, Northern Ireland, United Kingdom; Pinderfields Hospital, Yorkshire, United Kingdom, and Royal Hallamshire Hospital, Sheffield, United Kingdom; Leeds Teaching Hospitals National Health Service Trust, St James's University Hospital, Leeds, United Kingdom; Carol Davila University of Medicine and Pharmacy, Bucharest, Romania; Universidade de São Paulo, São Paulo, Brazil; Harvard Medical School, Massachusetts General Hospital, Boston, Massachusetts; School of Medicine and Biomedical Science, University of Sheffield, Sheffield, United Kingdom; Watford Hospital, Watford, United Kingdom; Leeds General Infirmary, Leeds, United Kingdom; University of Medicine and Pharmacy, Tirgu-Mures, Romania; Semmelweis University, Budapest, and Hungarian Academy of Sciences, Budapest, Hungary; Queen Margaret Hospital, Fife, United Kingdom; Newcastle University, Newcastle-upon-Tyne, United Kingdom; University of Milan, and Istituto Auxologico Italiano Istituto di Ricovero e Cura a Carattere Scientifico, Milan, Italy; University Medical Center Ljubljana, Ljubljana, Slovenia; Clinical Center of Serbia and Medical Faculty, University of Belgrade, Belgrade, Serbia; South Australia Pathology at the Women's and Children's Hospital, North Adelaide, South Australia, Australia; The National Hospital for Neurology and Neurosurgery, Queen Square, London, United Kingdom; Sir Charles Gairdner Hospital, Nedlands, West Australia, Australia; Institute of Genetic Medicine, University of Newcastle on Tyne, Royal Victoria Infirmary, Newcastle, United Kingdom; Barts and The London School of Medicine, Queen Mary University of London, London, United Kingdom; University of Warwick, Warwick, United Kingdom; Hospital das Clinicas, Minas Gerais Federal University, Belo Horizonte, Brazil; Wiinipeg University, Winnipeg, Canada; The John Radcliffe Hospital, Oxford, United Kingdom; Johns Hopkins University School of Medicine, Baltimore, Maryland; Universitätsklinikum Erlangen, Friedrich-Alexander-Universität, Erlangen-Nürnberg, Germany; Churchill Hospital, Oxford University Hospitals National Health Service Trust, Oxford, United Kingdom; Chelsea and Westminster Hospital National Health Service Foundation Trust, London, United Kingdom; Hospital das Clinicas, Minas Gerais Federal University, Belo Horizonte, Brazil; National Hospital of Sri Lanka, Colombo, Sri Lanka; Fondazione Cà Granda Istituto di Ricovero e Cura a Carattere Scientifico Ospedale Maggiore, University of Milan, Milan, Italy; Endocrine Clinic, San Clemente, California; The London Centre for Pediatric Endocrinology and Diabetes, Great Ormond Street Hospital for Children National Health Service Foundation Trust, London, United Kingdom; University Hospital Birmingham, and Birmingham Women's Hospital, Birmingham, United Kingdom; Barts and The London School of Medicine, Queen Mary University of London, London, United Kingdom; Charite Campus Mitte, Berlin, Germany; Santa Maria Nuova Hospital and Research Institute, Reggio-Emilia, Italy; Norfolk and Norwich University Hospital, Norwich, United Kingdom; University of Oxford, Oxford Centre for Diabetes, Endocrinology, and Metabolism, Churchill Hospital, Oxford, United Kingdom; Women's and Children's Hospital, Adelaide, Australia; Beaumont Hospital, Dublin, Republic of Ireland; University of Virginia, Charlottesville, Virginia; Faculty of Medicine, Semmelweis University, Budapest, Hungary; The Christie National Health Service Foundation Trust, Manchester, United Kingdom; Evangelismos Hospital, Athens, Greece; Evangelismos Hospital, Athens, Greece; Szolnok Hospital, Szolnok, Hungary; Great Western Hospitals National Health Service Foundation Trust, Swindon, United Kingdom; Institute of Endocrinology, Medical Academy, Lithuanian University of Health Sciences, Kaunas, Lithuania; Oxford Centre for Diabetes, Endocrinology, and Metabolism, Churchill Hospital, Oxford, United Kingdom; Hospital Sant Pau, Centre for Biomedical Research on Rare Diseases, (Centro de Investigación Biomédica en Red de Enfermedades Raras Unit 747); Universitat Autònoma de Barcelona, Barcelona, Spain; Liverpool Women's National Health Service Foundation Trust, Liverpool, United Kingdom; Toranomon Hospital, Tokyo, Japan; Istanbul University, Istanbul Faculty of Medicine, Istanbul, Turkey; The London Clinic, London, United Kingdom; Institute of Health Biosciences, The University of Tokushima Graduate School, Tokushima City, Japan; Royal Infirmary of Edinburgh, Edinburgh, Scotland, United Kingdom

## Abstract

**Context:**

Familial isolated pituitary adenoma (FIPA) due to aryl hydrocarbon receptor interacting protein (*AIP*) gene mutations is an autosomal dominant disease with incomplete penetrance. Clinical screening of apparently unaffected *AIP* mutation (*AIP*mut) carriers could identify previously unrecognized disease.

**Objective:**

To determine the *AIP* mutational status of FIPA and young pituitary adenoma patients, analyzing their clinical characteristics, and to perform clinical screening of apparently unaffected *AIP*mut carrier family members.

**Design:**

This was an observational, longitudinal study conducted over 7 years.

**Setting:**

International collaborative study conducted at referral centers for pituitary diseases.

**Participants:**

FIPA families (n = 216) and sporadic young-onset (≤30 y) pituitary adenoma patients (n = 404) participated in the study.

**Interventions:**

We performed genetic screening of patients for *AIP*muts, clinical assessment of their family members, and genetic screening for somatic *GNAS1* mutations and the germline *FGFR4* p.G388R variant.

**Main Outcome Measure(s):**

We assessed clinical disease in mutation carriers, comparison of characteristics of *AIP*mut positive and negative patients, results of *GNAS1*, and *FGFR4* analysis.

**Results:**

Thirty-seven FIPA families and 34 sporadic patients had *AIP*muts. Patients with truncating *AIP*muts had a younger age at disease onset and diagnosis, compared with patients with nontruncating *AIP*muts. Somatic *GNAS1* mutations were absent in tumors from *AIP*mut-positive patients, and the studied *FGFR4* variant did not modify the disease behavior or penetrance in *AIP*mut-positive individuals. A total of 164 *AIP*mut-positive unaffected family members were identified; pituitary disease was detected in 18 of those who underwent clinical screening.

**Conclusions:**

A quarter of the *AIP*mut carriers screened were diagnosed with pituitary disease, justifying this screening and suggesting a variable clinical course for *AIP*mut-positive pituitary adenomas.

Familial isolated pituitary adenoma (FIPA) is characterized by the presence of pituitary adenomas in two or more members of the same family in the absence of other syndromic clinical features, such as those characteristic of multiple endocrine neoplasia (MEN) type 1 and MEN4, Carney complex or tumors related to mutations in the succinate dehydrogenase genes. FIPA is a heterogeneous condition, encompassing cases with unknown genetic cause and patients with mutations in the aryl-hydrocarbon receptor interacting protein gene (*AIP*), with distinctive clinical characteristics. Germline *AIP* mutations (*AIP*muts) play a role not only in a subset of FIPA families (1–4) but also in sporadically diagnosed pituitary adenomas (5–9), and in the setting of somatostatin analog (SSA)-resistant acromegaly (10). Another form of FIPA, X-linked acrogigantism, due to microduplications in the Xq26.3 region, has been recently identified in patients with very young-onset gigantism and pituitary adenoma/hyperplasia (11).

The phenotype of *AIP*mut-associated pituitary adenomas has been described before (2–4, 12), but a systematic follow-up of cases and families is lacking due to the relative novelty of this pathogenic association (1), the variable disease penetrance (4, 12–14), and the rarity of this clinical entity. We present the clinical and genetic characteristics of a large cohort of FIPA and simplex (patients with germline mutation and no family history) *AIP*mut-positive patients, aiming for the following: 1) to perform a systematic follow-up of families to identify and characterize *AIP*mut-positive carriers, 2) to seek the role of disease-modifying genes on the variable phenotype and penetrance of the disease, and 3) to confirm and extend the description of the phenotype of *AIP*mut-positive patients, providing a comparison with *AIP*mut-negative cases. We establish that genetic screening followed by clinical assessment identifies a large percentage of family members with pituitary abnormalities, supporting the facilitation of genetic diagnosis and follow-up of these patients and their families.

## Patients and Methods

Our study population (1725 subjects, Table 1) was recruited via the collaborative research network of the International FIPA Consortium (15). Pituitary adenoma patients were grouped into 11 clinical diagnostic categories (Supplemental Table 1). The diagnoses of acromegaly, acromegaly/prolactinoma, gigantism, gigantism/prolactinoma, and mild acromegaly (16) were grouped together under the category of GH excess for some analyses.

**Table 1.** Study Population: Demographics and General Description

	Familial Cohort	Sporadic Cohort	Combined
Total individuals, n, %	1231 (71.4)	494 (28.6)	1725 (100)
Females, n, %	668 (54.3)	250 (50.6)	918 (53.2)
Current age, median (range [IQR])	46.2 (2–97 [32–62])	35 (3–77 [26–42])	42.6 (2–97 [29–56])
Clinical status, n, %			
Affected	502 (40.8)	404 (81.8)	906 (52.5)
Unaffected	729 (59.2)	90 (18.2)	819 (47.5)
Affected males, n, %	219 (43.6)	203 (50.2)	422 (46.6)
Affected females, n, %	283 (56.4)	201 (49.8)	484 (53.4)
Diagnoses, n, %			
Acromegaly	170 (33.9)	203 (50.2)	373 (41.2)
Acromegaly/prolactinoma	17 (3.4)	12 (3)	29 (3.2)
Cushing's disease	24 (4.8)	21 (5.2)	45 (5)
FSHoma	2 (0.4)	1 (0.2)	3 (0.3)
Gigantism	44 (8.8)	65 (16.1)	109 (12)
Gigantism/prolactinoma	1 (0.2)	10 (2.5)	11 (1.2)
Mild acromegaly	2 (0.4)	—	2 (0.2)
NFPA	91 (18.1)	21 (5.2)	112 (12.4)
Pituitary tumor	17 (3.4)	2 (0.5)	19 (2.1)
Prolactinoma	134 (26.7)	67 (16.6)	201 (22.2)
TSHoma	—	2 (0.5)	2 (0.2)
GH excess patients, n, %	234 (46.6)	290 (71.8)	524 (57.8)

Abbreviations: FSHoma, FSH secreting adenoma. TSHoma, thyrotropinoma.

Dash indicates no patients in this category.

Between January 2007 and January 2014, we recruited patients from 35 countries from two different groups: either members of FIPA families, defined by the presence of pituitary adenomas in two or more members of a family without other associated clinical features (1–3, 17) (familial cohort), or sporadically diagnosed pituitary adenoma patients with disease onset at 30 years of age or younger (sporadic cohort). As an exception to these inclusion criteria, one *AIP*mut-positive sporadic patient older than 30 years was found thanks to *AIP* screening in the setting of a research study, and the screening of his relatives detected a second *AIP*mut-positive pituitary adenoma case; this family was included in the familial cohort. The first patient reported in each FIPA family and all the sporadic patients were considered probands. All the patients received treatment and were followed up in accordance with the guidelines and clinical criteria of their respective centers. Relevant clinical and family structure data were received from clinicians and/or patients, and genetic screening was performed in the families of all the *AIP*mut-positive probands, selecting individuals according to their risk of inheriting the mutation, based on their position in the family tree, and extending the screening to as many generations as possible. In both familial and sporadic cases, other causes of familial pituitary adenomas, such as MEN1 and MEN4, Carney complex, pheochromocytoma/paraganglioma and pituitary adenoma syndrome, and X-linked acrogigantism were ruled out by clinical, biochemical and, in some cases, genetic tests, as appropriate.

The study population included a great majority of new cases but also previously diagnosed patients being followed up by the participating centers and a few historical cases, corresponding to deceased members of FIPA families (further details in Supplemental Results). Four *AIP*mut-positive patients (two with diagnosis of acromegaly and two with gigantism) died in the postrecruitment period. Three of the deaths were due to cardiovascular causes (stroke, chronic heart failure, and acute coronary syndrome), whereas the exact cause of death is unknown in the fourth, a patient with long-standing untreated familial acromegaly.

All the patients and family members included agreed to take part by providing signed informed consent forms approved by the local ethics committee. Further details on the study population and the procedures for genetic/clinical screening and search for disease-modifying genes are described in the Supplemental Material.

### Statistical analysis

The qualitative, categorical variables were expressed as percentages and compared using the χ^2^ test or the Fisher's exact test, as appropriate. The normal distribution of the quantitative variables was verified using the Shapiro-Wilk and the Kolmogorov-Smirnov tests for normality. Means and SDs were used to report parametric data, and nonparametric data were expressed as median and interquartile ranges. Parametric data were analyzed with the unpaired *t* test, with a 95% confidence interval (CI), whereas the Mann-Whitney *U* test was used for the nonparametric data. Statistical significance was considered when the *P* value was < .05. All the statistical analyses were carried out using the GraphPad Prism 6 (GraphPad Software Inc) and Stata 12 (StataCorp LP) statistical software.

## Results

### Study population

The familial cohort was composed of 216 FIPA families, including 156 new families (989 subjects: 337 patients and 652 unaffected family members) and 60 previously described families (3, 12), in which 46 new subjects (15 patients and 31 unaffected family members) were added to the previously reported 196 individuals (150 patients and 46 unaffected family members). The sporadic cohort originally included 409 pituitary adenoma patients 30 years old or younger at disease onset, with no known family history of pituitary adenoma, but we excluded five patients from further analysis due to harboring an Xq26.3 microduplication. Of the remaining 404 sporadic patients, six were reported previously (3). In addition to the *AIP*mut screening, a subset of *AIP*mut-negative FIPA (n = 55) and sporadic (n = 45) patients underwent genetic screening for other endocrine neoplasia-associated genes (Supplemental Table 2). All of these tests were negative for pathogenic variants. After the genetic screening and follow-up of the patients and carriers, 60 individuals in the familial cohort and seven in the sporadic cohort were classified as not at risk of inheriting an *AIP*mut and were excluded from further analysis. Twenty-three individuals initially thought to be unaffected were identified with pituitary abnormalities (see details in *Prospective diagnosis*).

### Genetic screening results

Thirty-seven of 216 FIPA families screened (17.1%) and 34 of 404 sporadic patients (8.4%) were positive for pathogenic or likely pathogenic *AIP*muts, accounting for a total of 71 *AIP*mut-positive kindreds and 144 *AIP*mut-positive patients (76.4% familial and 23.6% simplex, Table 2). We also identified 164 *AIP*mut-positive apparently unaffected family members (see *Follow-up and prospective diagnosis*). Samples were not available from family members of 25 *AIP*mut-positive simplex cases to establish the presence or lack of de novo mutations. We identified three pituitary adenoma patients (two with clinically nonfunctioning pituitary adenoma [NFPA] and one with a microprolactinoma) belonging to *AIP*mut-positive FIPA families and being at risk of inheriting but not carrying an *AIP*mut; therefore, they were considered as phenocopies.

**Table 2.** Screening for *AIP*muts

	Familial Cohort			Sporadic Cohort			Combined		
	*AIP*mut-Positive Familial	*AIP*mut-Negative Familial	Total Familial	*AIP*mut-Positive Simplex	*AIP*mut-Negative Sporadic	Total Sporadic	*AIP*mut-Positive Familial and Simplex	*AIP*mut-Negative Familial and Sporadic	Total
Total number of kindreds, n, %	37 (17.1% of familial)	179 (82.9% of familial)	216 (34.8% of total)	34 (8.4% of sporadic)	370 (91.6% of sporadic)	404 (65.2% of total)	71 (11.5% of total)	549 (88.5% of total)	620 (100)
Total individuals, n, %	475 (38.6% of familial)	756 (61.4% of familial)	1231 (71.4% of total)	82 (16.6% of sporadic)	412 (83.4% of sporadic)	494 (28.6% of total)	557 (32.3% of total)	1168 (67.7% of total)	1725 (100)
Genetic status, n, %									
*AIP*mut-negative patients	3 (0.6)	389 (51.5)^a^	392 (31.8)	—	370 (89.8)	370 (74.9)	3 (0.5)	759 (65)	762 (44.2)
*AIP*mut-positive tested patients	95 (20)	—	95 (7.7)	34 (41.5)	—	34 (6.9)	129 (23.2)	—	129 (7.5)
At risk but not tested	33 (6.9)	—	33 (2.7)	8 (9.8)	—	8 (1.6)	41 (7.4)	—	41 (2.4)
Not at risk	48 (10.1)	12 (1.6)	60 (4.9)	7 (8.5)	—	7 (1.4)	55 (9.9)	12 (1)	67 (3.9)
Obligate unaffected carriers, not tested	8 (1.7)	—	8 (0.6)	2 (2.4)	—	2 (0.4)	10 (1.8)	—	10 (0.6)
Predicted *AIP*mut-positive patients	15 (3.2)	—	15 (1.2)	—	—	—	15 (2.7)	—	15 (0.9)
Unaffected *AIP*mut tested carriers	120 (25.3)	—	120 (9.7)	16 (19.5)	—	16 (3.2)	136 (24.4)	—	136 (7.9)
Unaffected and *AIP*mut negative	153 (32.2)	—	153 (12.4)	15 (18.3)	—	15 (3)	168 (30.2)	—	168 (9.7)
Unaffected relatives of *AIP*mut-negative patients	—	355 (47)	355 (28.8)	—	42 (10.2)	42 (8.5)	—	397 (34)	397 (23)
Summary of *AIP*mut-positive individuals, n, %									
Total *AIP*mut-positive patients^b^	110 (23.2)	—	110 (8.9)	34 (41.5)	—	34 (6.9)	144 (25.9)	—	144 (8.3)
Total unaffected *AIP*mut carriers^c^	128 (26.9)	—	128 (10.4)	18 (22)	—	18 (3.6)	146 (26.2)	—	146 (8.5)

Dash indicates no individuals in this category.

a In *AIP*mut-negative FIPA families, 199 patients were tested for *AIP*muts; the rest (n = 190) were assumed to be negative.

b This is equal to the sum of tested *AIP*mut-positive patients plus the predicted *AIP*mut-positive patients.

c Sum of tested unaffected carriers plus obligate unaffected carriers.

Thirty-one different *AIP*muts (10 not previously reported) were identified in our study population: 12 exclusively in familial cases, 12 in simplex cases only, and seven in both settings (Table 3 and Supplemental Figure 1). Of the total mutations, 70.8% (22/31) predicted a truncated or missing protein and were termed as truncating *AIP*muts (Supplemental Figure 2). We also identified 11 apparently nonpathogenic *AIP* variants (three of them novel) in our population (Supplemental Table 3).

**Table 3.** *AIP* Pathogenic or Likely Pathogenic Mutations in the Familial and Sporadic Cohorts

Mutation (DNA Level [Protein Level])	Mutation Type	Pathogenic	Location in Protein	Familial Cohort (n = 238)^a^	Simplex Cohort (n = 52)^a^	Combined (n = 290)^a^	References/SR^b^
g.4856_4857CG>AA	Promoter	Yes^c^	Not in protein (5′ UTR)	3 (1.3)	—	3 (1)	(3, 12)/(SR30)
c.3G>A (p.?)	Start codon	Likely^c^	N terminus	2 (0.8)	—	2 (0.7)	This paper
c.40C>T (p.Q14*)	Nonsense	Yes^c^	N terminus	2 (0.8)	—	2 (0.7)	(1)/(SR31, 32)
c.70G>T (p.E24*)	Nonsense	Yes^c^	N terminus	9 (3.8)	—	9 (3.1)	(3)/(SR33)
c.74_81delins7 (p.L25Pfs*130)	Frameshift	Yes^c^	PPIase domain	10 (4.2)	—	10 (3.4)	(12)/(SR34)
c.100–1025_279 + 357del (ex2del) (p.A34_K93del)	Large genomic deletion	Yes^c^	PPIase domain	12 (5)	2 (4)	14 (4.8)	(SR35)
c.100–18C>T	Intronic	Likely	Not in protein (intron 1)	—	3 (6)	3 (1)	(3, 7, 10)/(SR31)
c.241C>T (p.R81*)	Nonsense	Yes^c^	PPIase domain	12 (5)	4 (8)	16 (5.5)	(3)/(SR30, 36–38)
c.249G>T (p.G83Afs*15)	Splice site (cryptic splice site)	Yes^c^	PPIase domain	4 (1.7)	—	4 (1.4)	(12)
c.338_341dup (p.L115Pfs*16)	Frameshift	Yes^c^	PPIase domain	—	2 (4)	2 (0.7)	(6)
c.427C>T (p.Q143*)	Nonsense	Yes^c^	Between PPIase and TPR1 domains	—	1 (2)	1 (0.3)	This paper
c.469–2A>G (p.E158_Q184del)	Splice site	Likely	TPR1 domain	—	1 (2)	1 (0.3)	(5)/(SR39, 40)
c.490C>T (p.Q164*)	Nonsense	Yes^c^	Between PPIase and TPR1 domains	3 (1.3)	—	3 (1)	(12)
c.570C>G (p.Y190*)	Nonsense	Yes^c^	TPR1 domain	9 (3.8)	—	9 (3.1)	This paper
c.662dupC (p.E222*)	Nonsense	Yes^c^	Between TPR1 and 2 domains	3 (1.3)	—	3 (1)	(12)
c.713G>A (p.C238Y)	Missense	Yes	TPR2 domain	4 (1.7)	—	4 (1.4)	(3)/(SR33)
c.783C>G (p.Y261*)	Nonsense	Yes^c^	TPR2 domain	4 (1.7)	—	4 (1.4)	(9)/(SR39, 41, 42)
c.787 + 9C>T	Intronic	Uncertain	Not in protein (intron 5)	—	1 (2)	1 (0.3)	This paper
c.804C>A (p.Y268*)	Nonsense	Yes^c^	TPR3 domain	19 (8)	3 (6)	22 (7.6)	(SR43, 44)
c.805_825dup (p.F269_H275dup)	In-frame insertion	Yes	TPR3 domain	16 (6.7)	2 (4)	18 (6.2)	(3)/(SR30, 39, 45)
c.807C>T (p.(=))	Splice site (reduced transcript level)	Yes	TPR3 domain	7 (2.9)	4 (8)	11 (3.8)	(3, 5, 7, 10, 12)/(SR46, 47)
c.811C>T (p.R271W)	Missense	Yes	TPR3 domain	—	1 (2)	1 (0.3)	(2, 7, 12)/(SR48)
c.816delC (p.K273Rfs*30)	Frameshift	Yes^c^	TPR3 domain	—	1 (2)	1 (0.3)	This paper
c.868A>T (p.K290*)	Nonsense	Yes^c^	TPR3 domain	—	1 (2)	1 (0.3)	This paper
c.872_877delTGCTGG (p.V291_L292del)	In-frame deletion	Yes	TPR3 domain	—	1 (2)	1 (0.3)	This paper
c.910C>T (p.R304*)	Nonsense	Yes^c^	C-terminal α-helix	88 (37)	16 (31)	104 (35.9)	(1–3, 5, 7, 9, 12, 14)/ (SR39, 49–51)
c.911G>A (p.R304Q)	Missense	Yes	C-terminal α-helix	20 (8.4)	3 (6)	23 (7.9)	(3, 5, 7, 9, 12)/(SR31, 39, 52, 53)
c.967delC (p.R323Gfs*39)	Frameshift	Yes^c^	C-terminal α-helix	—	4 (8)	4 (1.4)	This paper
c.976_977insC (p.G326Afs*?)	Frameshift	Yes^c^	C-terminal α-helix	—	1 (2)	1 (0.3)	This paper
c.978dupG (p.I327Dfs*?)	Frameshift	Yes^c^	C-terminal α-helix	—	1 (2)	1 (0.3)	This paper
c.1-?_993+?del− (whole gene deletion)	Large genomic deletion	Yes^c^	Absence of the whole protein	11 (4.6)	—	11 (3.8)	(12)

Abbreviations: PPIase, peptidylprolyl isomerase; SR, supplemental references; TPR, tetratricopeptide repeat; UTR, untranslated region.

Dash indicates no individuals in this category.

a Number of positive individuals for each mutation, considering the *AIP*mut-positive tested individuals, the obligate carriers, and the predicted *AIP*mut patients.

b For supplemental references, see Supplemental Material.

c Truncating mutation.

A multiple regression analysis was performed to determine which clinical features could more accurately predict the likelihood of a patient to carry an *AIP*mut. An age at diagnosis of 10 years or older and younger than 20 years conferred an odds ratio (OR) of 5.8 (*P* = .000, 95% CI 3.1–10.8) of having an *AIP*mut, whereas the OR was 2.8 if the age at diagnosis was 20 years or older and younger than 30 years (*P*= .000, 95% CI 1.3–5.7); thus, an age at diagnosis between 10 and 30 years is the best predictor of *AIP*muts. Inversely, a diagnosis of prolactinoma resulted in an OR of 0.2 (*P*= .000, 95% CI 0.1–0.5).

### Genotype-phenotype correlation within the *AIP*mut-positive cohort

**Figure 1.**
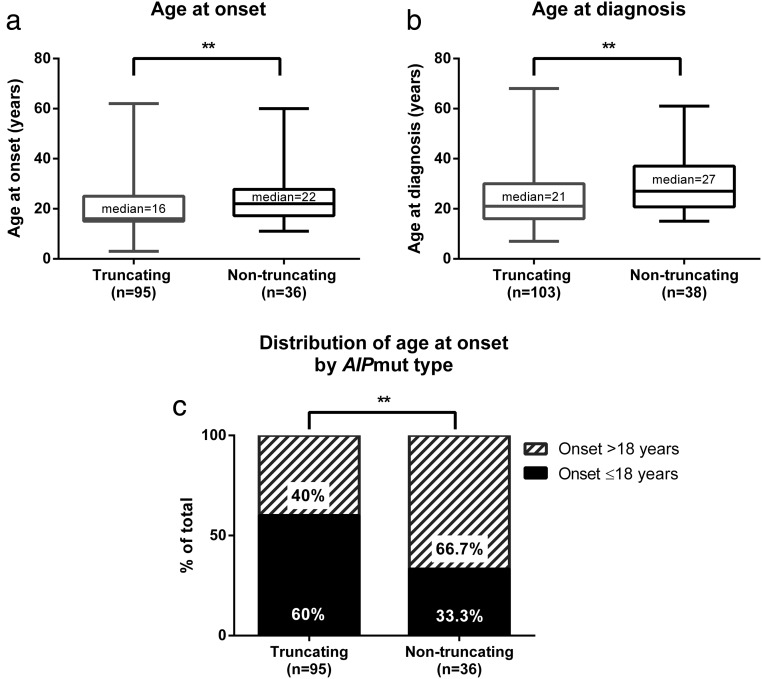
Patients with truncating vs nontruncating *AIP*muts. Patients with truncating *AIP*muts present with a more aggressive phenotype, characterized by an earlier age at onset (A) (*P* = .005) and (B) at diagnosis (*P* = .003). C, This earlier disease onset results in a higher frequency of pediatric cases (n [total] = 131); in fact, most of the patients with truncating mutations present in childhood and adolescence. **, *P* < .01.

Truncating mutations accounted for 78.9% of the *AIP*muts found in the familial cohort (15 of 19) and for 57.9% of those detected in the sporadic cohort (11 of 19). To study a possible difference in disease penetrance between truncating and nontruncating mutations, we compared the number of affected individuals with truncating *AIP*muts in the familial (85 of 110 [77.3%]) and simplex cohorts (21 of 34 [61.8%]), finding no significant difference, although a trend was observed (*P* = .0729, analysis included prospectively diagnosed patients). No significant differences were found regarding the proportion of GH excess cases, number of patients per family, maximum tumoral diameter, frequency of macroadenomas, extrasellar invasion, or number of treatments received between the patients with truncating and nontruncating mutations. However, patients with truncating mutations were significantly younger at disease onset (median 16 [interquartile range (IQR) 15–25] vs 22 [IQR 17.3–27.8] y, *P* = .0046, Figure 1A) and at diagnosis (median 21 [IQR 16–30] vs 27 [IQR 20.8–37] y, *P* = .0028, Figure 1B), and the occurrence of pediatric cases was more common in this group (60% [57 of 95], Figure 1C), compared with the patients with nontruncating *AIP*muts (33.3% [12 of 36], *P* = .0064). In concordance with these differences, gigantism accounted for a significantly higher proportion of the GH excess cases in the patients with truncating *AIP*muts (54.7% [47 of 86]), compared with those with nontruncating *AIP*muts (30% [9 of 30], *P* = .0200). Because p.R304* was the most common *AIP*mut in our study population (20 kindreds), we analyzed whether these patients behaved differently from other patients with truncating mutations. We found more affected individuals per family (median 4 [IQR 2.5–5]) among families carrying the p.R304* *AIP*mut, compared with families with other *AIP*muts (median 2 [IQR 2–3], *P* = .0133). When considering all the *AIP*mut-positive patients together (familial and sporadic), we found a higher proportion of pediatric patients among those with the *AIP* p.R304* mutation (65.8% [25 of 38] vs 46.5% [40 of 86], *P* = .0475).

### Clinical and histopathological features

Findings regarding gender distribution, age at onset/diagnosis, distribution of clinical diagnoses, tumor size/extension, pituitary apoplexy, histopathological features, extrapituitary tumors, and specific analyses of patients with GH excess and with gigantism are detailed in the Supplemental Material and depicted in Supplemental Tables 4 and 5 and Supplemental Figures 3–7.

### Disease penetrance

To calculate the penetrance of pituitary adenomas among *AIP*mut positive families, complete data are needed both for phenotype and genotype. Therefore, we have selected three families (two with p.R304* and one with p.A34_K39del mutations) in which complete data were available in three or more generations for consenting at-risk individuals. The *AIP* genotype was known in 76.6% (range 68.4%–94.7%) of the individuals at risk; of them, 16.8% were patients and 83.2% were unaffected carriers. The gender distribution was similar between patients and unaffected carriers. The mean penetrance in these three families was 28.6% (19%–38.1%), and it decreased to 22.7% (18.2%–26.7%) when 50% of the individuals at risk with unknown genotype were considered as unaffected carriers. When the prospectively diagnosed patients were omitted from this calculation, the total penetrance of pituitary adenomas was 12.5%, highlighting the importance of the follow-up of apparently unaffected carriers for the correct calculation of the disease penetrance.

Because penetrance cannot be appropriately calculated for *AIP*mut-negative families, we assessed the number of affected family members. The *AIP*mut-positive families had more affected individuals per family than the *AIP*mut-negative families (*P* < .0001, Supplemental Figure 7E). Whereas 84.9% of the *AIP*mut-negative families (152 of 179) had only two affected members, 48.6% of the *AIP*mut-positive families (18 of 37) had three or more pituitary adenoma patients per family. The maximum number of affected individuals within the same family was eight (six of them prospectively diagnosed) in a family carrying the p.R304* *AIP*mut, and the maximum number of cases of gigantism in the same family was five, in a FIPA family with the p.E24* *AIP*mut.

### Follow-up and prospective diagnosis

Of the 164 originally identified *AIP*mut carriers, 160 were available and advised to undergo biochemical and clinical screening. Prospective diagnosis of a pituitary adenoma was established in 11.3% (18 subjects, 11 males) of the individuals originally considered as unaffected *AIP*mut carriers.

Six of the prospectively diagnosed patients had acromegaly (one of them with prolactin [PRL] cosecretion), one patient had gigantism, two patients were diagnosed with mild acromegaly (16), and nine patients harbored NFPAs. Of the 142 individuals remaining as apparently unaffected *AIP*mut carriers, 79 (55.6%) underwent clinical assessment and one or more biochemical or imaging tests, whereas 63 subjects (44.4%) had only clinical evaluation.

The prospective cases were diagnosed at an older age than the rest of the patients (median 30 [IQR 22.8–39.5] vs 23 [IQR 16–33] y, *P* = .025). At diagnosis, seven of the prospectively diagnosed patients were symptomatic (headaches, arthralgias, acral growth, facial changes, weight gain, or hyperhidrosis). Five of the 18 prospectively diagnosed tumors were macroadenomas, in contrast with a predominance of macroadenomas (89.9%, 71 of 79) in the rest of the *AIP*mut-positive FIPA patients (*P* < .0001). The maximum diameter was significantly smaller for prospective cases (median 5.8 [IQR 4.7–14.4] vs 16.5 [IQR 10–29] mm, *P* = .0002). Four of the patients with macroadenomas had surgery, and the histopathological study confirmed GH- or GH/PRL-positive adenomas. The fifth macroadenoma was identified in a 68-year-old male patient with high IGF-1, well-controlled hypertension and diabetes mellitus and no other comorbidities or symptoms, who did not want to receive any treatment. In addition, one *AIP*mut-negative pituitary adenoma patient, harboring a 25-mm NFPA, was prospectively diagnosed as part of an *AIP*mut-positive family (brother of the *AIP*mut positive proband).

A further seven subjects had abnormalities in their screening tests, but a pituitary disease was not confirmed: five individuals had slightly elevated IGF-1 levels for their age/gender, one patient displayed acromegaloid features but normal pituitary magnetic resonance imaging (MRI) and biochemistry, and a 17-year-old female had repeatedly borderline high IGF-1 and incompletely suppressed GH on oral glucose tolerance test, but her bulky pituitary gland (11 mm in height), normal at this age group, is not changing during follow-up and her biochemical results are now within the normal range, after 3 years of follow-up.

**Figure 2.**
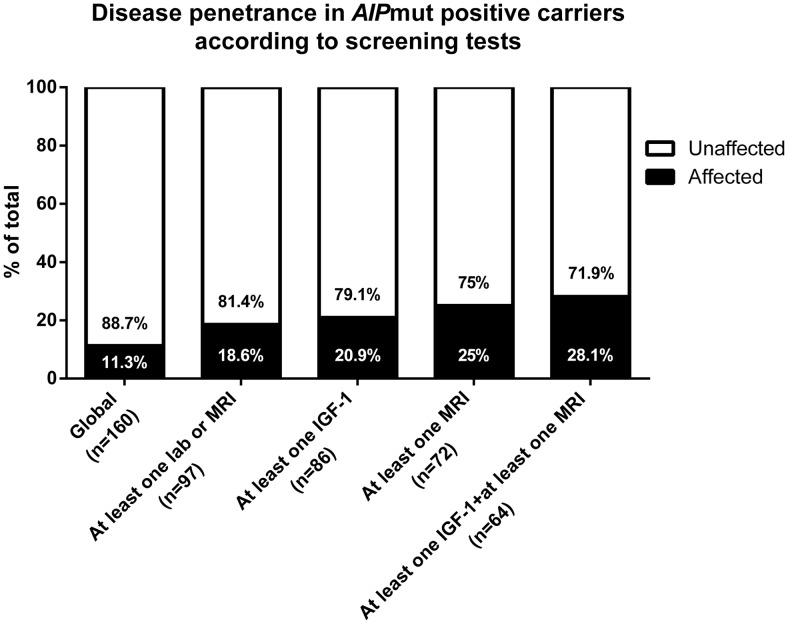
Penetrance in screened *AIP*mut-positive carriers (n [total] = 160). The probability of detecting new cases of pituitary adenomas within apparently unaffected *AIP*mut carriers depends on the clinical assessment and the type of complementary biochemical/imaging studies included in the screening protocol (see text).

The global penetrance of pituitary adenomas among the individuals initially considered as unaffected *AIP*mut carriers was 11.3% (18 of 160). However, the penetrance was higher in the group of carriers who underwent biochemical and imaging investigations, varying between 18.6% and 28.1%, depending on the depth of screening (Figure 2). Overall, these data suggest that approximately 20%–25% of the apparently unaffected *AIP*mut carriers screened with biochemical or imaging tests will be identified with a pituitary adenoma at some point in their lives.

Clinical screening was not systematically performed in the *AIP*mut-negative FIPA unaffected family members. Nevertheless, due to the increased disease awareness given by the existence of previous pituitary adenoma cases within their families, four individuals (three females and one male) from three different *AIP*mut-negative FIPA families were prospectively diagnosed. Three of them harbored NFPAs, but we lack complete information about the fourth patient. The mean age at diagnosis in the three NFPA cases was 37 years, and only one patient referred symptoms at diagnosis (galactorrhea, not clearly associated to stalk compression, and lethargy). All of them had microadenomas, with a mean diameter of 6.5 mm and did not require any therapeutic intervention other than hormonal replacement in one case. The characteristics of these cases resemble those of incidentalomas; however, the occurrence of two prospective cases in the same family supports an apparent inherited component.

### Disease-modifying genes

We have studied the role of two possible disease-modifying genes: *GNAS1* (somatic) (18) and *FGFR4* (germline) (19). *GNAS1* mutations were absent in all the studied *AIP*mut-positive somatotropinomas (n = 23) but were detected in 50% of the *AIP*mut-negative familial somatotropinomas (5 of 10), 16.7% of the *AIP*mut-negative, young-onset cases (1 of 6), and 26.3% of the unselected acromegaly cases studied (5 of 19). The distribution of the *FGFR4* p.G388R single-nucleotide polymorphism (SNP) conserved the Hardy-Weinberg equilibrium (20), and the genotype distribution was similar between patients (n = 98) and *AIP*mut carriers (n = 108) (*P* = .523). The age at onset and at diagnosis, tumor size, and frequency of extrasellar invasion were not significantly different between the GG (wild type) and GR/RR patients.

## Discussion

*AIP*muts are prevalent in young-onset, GH excess patients (24%) and FIPA (17.1%), with more than double frequency in patients with gigantism (46.7%) in our cohort, in concordance with other studies (7, 9, 21, 22). However, in contrast to previous reports, in this large and extensively studied cohort, there was no predominance of male patients among the *AIP*mut-positive familial cases, and equal numbers of male and female unaffected carriers were identified. Earlier studies (3, 4, 12, 23) may have had an ascertainment bias for families with cases of gigantism, a disease that is more prevalent in males, at least partly due to the physiologically later puberty and therefore later cessation of growth in boys.

We have demonstrated that approximately a quarter of the individuals initially identified as unaffected *AIP*mut carriers who underwent clinical screening tests were diagnosed with pituitary abnormalities. Full clinical screening identified 28.1% of the carriers, with fewer tests understandably resulting in fewer positive cases. Our data suggest that not all the *AIP*mut-associated pituitary adenomas have a rapidly growing, aggressive phenotype. The follow-up of these patients allowed us to observe some probably very early cases of acromegaly, in which the current clinical scenario had not indicated intervention at data closure. We cannot rule out that some of the small NFPAs are indeed incidentalomas, similar to those frequently observed in *AIP*mut-negative subjects of the general population.

This frequency of prospective diagnosis may justify the clinical screening and, possibly, follow-up of all the *AIP*mut-positive unaffected carriers. Our data would support the assessment of all the newly identified *AIP*mut carriers (clinical examination/history, PRL, and IGF-1, as a minimum, up to a full screening, also including an oral glucose tolerance test and contrast enhanced pituitary MRI). Follow-up of the younger family members should continue until at least 30 years of age, preferably annually, with clinical assessment and basal pituitary hormonal levels, leaving stimulation tests for cases with suspicion of pituitary disease and a follow-up MRI if necessary (24, 25). The cost-effectiveness and the possible psychological burden of this approach will need future study. Stopping the follow-up should be considered in older patients, given the low possibility of detecting new pituitary adenoma patients in these individuals after the fifth decade of life (24, 25). Once a case has been prospectively diagnosed, the treatment and follow-up should proceed as for the general population of pituitary adenoma patients because there are no data to suggest a different type of treatment in *AIP*mut-positive patients (26) although reduced SSA responsiveness has been described.

The genetic and clinical screening of *AIP*mut-negative FIPA families is uncertain at this point. Baseline screening and follow-up of obligate carriers could be considered, keeping in mind that the age of onset is considerably older in these families. Education on possible signs and symptoms of family members is a viable option in the routine setting. We expect that the identification of further genes implicated in the pathogenesis of FIPA in the next years will allow us to tailor these recommendations in accordance with the clinical behavior of each genetic entity.

Patients with GH excess starting before the age of 5 years should be tested for the recently identified Xq26.3 chromosomal microduplications (11). The genetic screening of other sporadic, young-onset pituitary adenoma patients with no evidence of other endocrine tumors should be focused on *AIP*muts in first instance in cases of GH excess (with or without PRL cosecretion) and on *MEN1* mutations, especially in cases of prolactinoma (9) because this can be the first manifestation of MEN1 (27). Whether it would be advisable to continue screening young patients with other diagnoses for *AIP*muts out of the setting of research studies needs longer follow-up.

To explain the variable clinical phenotype in our *AIP*mut-positive patients, we evaluated the possible influence of two disease-modifying genes, *GNAS1* and *FGFR4*. Whereas somatic *GNAS1* mutations are common in unselected somatotropinomas (4.4%–59% of the cases) (28–35), we have not identified any in adenomas from *AIP*mut-positive patients, suggesting that germline *AIP*muts and somatic *GNAS1* mutations are mutually exclusive in somatotropinomas. *GNAS1* mutations have rarely been studied in pediatric patients with acromegaly and gigantism, and they seem to be an extremely infrequent finding in this age group (36, 37). A recent study has shown no change in the AIP immunostaining in sporadic somatotropinomas in the presence of *GNAS1* mutations (38). The characteristic phenotype of adenomas containing the *GNAS1* mutations (small [32, 39], highly responsive to the treatment with SSAs, and more often densely granulated according to some [40], but not all studies [41]), seems to be in contrast with the typical *AIP*mut-positive tumor phenotype. On the other hand, in somatotroph adenomas of *AIP*mut-negative FIPA patients, half of the tested samples had *GNAS1* mutations. This suggests that in *AIP*mut-negative FIPA, the somatic *GNAS1* mutations could exist in a similar frequency as in unselected somatotropinomas and possibly, in addition to a germline predisposing mutation, may play a role in their pathogenesis.

The *FGFR4* gene SNP rs351855 (c.1162G>A, p.G388R), with a minor allele frequency of 0.3, is a predictor of progression and poor prognosis in a variety of human neoplasms (42). A role for rs351855 as a facilitator of somatotroph cell tumorigenesis has been recently proposed (19), and we hypothesized that this variant could increase the penetrance and/or size and extension of *AIP*mut-positive pituitary adenomas. The screening for this SNP in our *AIP*mut-positive patients failed to show increase in size, extension, or apoplexy, even though this association had previously been suggested in sporadic acromegaly patients (19), and no earlier onset or higher penetrance was observed. The lack of association with these two potentially disease-modifying genes suggests that *AIP*mut-related pituitary adenomas are regulated by different pathogenic mechanisms than unselected somatotropinomas.

We recognize the numerous limitations of our study. We chose an arbitrary age cutoff (≤30 y), in concordance with previous *AIP*-related publications, but our data show that only 13.2% of the *AIP*mut-positive patients had disease onset after the age of 30 years. Our patients were recruited from different genetic backgrounds, and this could have influenced the disease penetrance and presentation. On the other hand, 19.7% of the *AIP*mut-positive kindreds (24.3% of the *AIP*mut positive patients) belong to a cohort with a founder *AIP*mut (p.R304*), originally from Northern Ireland (14). The larger number of subjects screened in these families provided a higher number of carriers and chance for detection of affected individuals. Additional genetic traits possibly cosegregating with this founder mutation could modify the phenotype and thus introduce a bias into our results. Full genotype and phenotype data were not available for all the families; therefore, we limited our penetrance calculations to three large, well-characterized families. A better assessment of the prevalence of pituitary apoplexy and extrapituitary adenomas in *AIP*mut-positive patients would require a large control group, screened ad hoc, which was beyond the scope of this study. Finally, the data about therapeutic modalities were limited, hampering the analysis of the response to different treatments.

### Conclusions

The analysis of this large cohort of FIPA patients allowed us to establish a number of novel aspects of FIPA. A phenotype-genotype correlation was found with younger onset of disease in patients with truncating *AIP*muts. We identified a surprisingly high percentage of somatic *GNAS1* mutations in the *AIP*mut-negative somatotropinomas and their absence in *AIP*mut-positive tumors. The lack of influence of the germline *FGFR4* p.G388R variant on disease penetrance/severity suggests that currently unknown factors drive penetrance and variable phenotype in *AIP*mut-positive pituitary adenomas. The presence of milder, more indolent disease in some *AIP*mut-positive subjects has been established. Genetic and clinical screening leads to the prospective identification of an unexpectedly high proportion of affected patients in the originally apparently unaffected carrier group, resulting in earlier diagnosis and treatment and, possibly, better long-term outcome (25). The recruitment of a large study population with this uncommon disease has only been possible thanks to worldwide collaboration.
